# Investigating the network consequences of focal brain lesions through comparisons of real and simulated lesions

**DOI:** 10.1038/s41598-021-81107-9

**Published:** 2021-01-26

**Authors:** Yuan Tao, Brenda Rapp

**Affiliations:** Department of Cognitive Science, Johns Hopkins University, Baltimore, USA

**Keywords:** Diseases of the nervous system, Stroke, Network models

## Abstract

Given the increased interest in the functional human connectome, a number of computer simulation studies have sought to develop a better quantitative understanding of the effects of focal lesions on the brain’s functional network organization. However, there has been little work evaluating the predictions of this simulation work vis a vis real lesioned connectomes. One of the few relevant studies reported findings from real chronic focal lesions that only partially confirmed simulation predictions. We hypothesize that these discrepancies arose because although the effects of focal lesions likely consist of two components: *short-term node subtraction* and *long-term network re-organization*, previous simulation studies have primarily modeled only the short-term consequences of the subtraction of lesioned nodes and their connections. To evaluate this hypothesis, we compared network properties (*modularity, participation coefficient, within-module degree*) between real functional connectomes obtained from chronic stroke participants and “pseudo-lesioned” functional connectomes generated by subtracting the same sets of lesioned nodes/connections from healthy control connectomes. We found that, as we hypothesized, the network properties of real-lesioned connectomes in chronic stroke differed from those of the pseudo-lesioned connectomes which instantiated only the short-term consequences of node subtraction. Reflecting the long-term consequences of focal lesions, we found re-organization of the neurotopography of global and local hubs in the real but not the pseudo-lesioned connectomes. We conclude that the long-term network re-organization that occurs in response to focal lesions involves changes in functional connectivity within the remaining intact neural tissue that go well beyond the short-term consequences of node subtraction.

## Introduction

Along with the very rapid increase in graph-theoretic approaches to the analysis of healthy connectome data (structural and functional), there have been increasing applications of this approach to investigating the neural underpinning of various brain diseases, such as stroke (e.g.^1–3^), schizophrenia (e.g.^4–6^), and Alzheimer’s disease (e.g.^7–10^). In addition, these approaches have also been applied to understanding the neuroplasticity that supports recovery of function after neural damage^11–13^; for reviews also see^14–16^. In the context of focal lesions, a number of computer simulation studies have been directed at developing a better quantitative understanding of the effects of focal lesions on the brain’s network organization. However, little work has been done to evaluate whether the predictions of this simulation work are observed in empirical data (for a review, see^15^). In fact, in one of the few studies that report relevant data, Gratton et al.^2^ presented findings from real focal lesions that only partially confirm the simulation predictions. In the current investigation, we first replicate the Gratton et al.^2^ findings and then carry out further analyses to test our hypotheses that these discrepancies arise due to long-term functional re-organization which is not captured by traditional simulation approaches.

### Discrepancies between simulated and real lesioned networks

**Figure 1:**
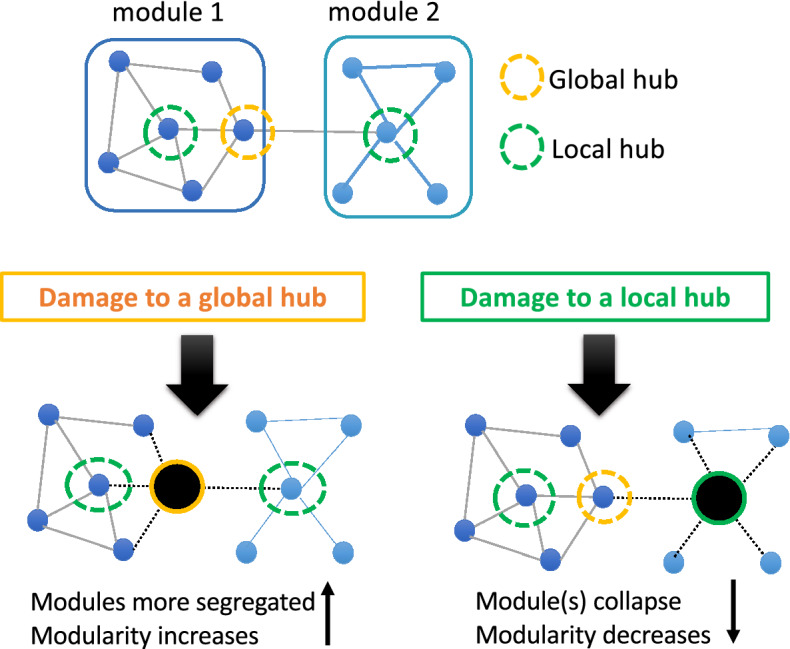
A theoretical framework for understanding the effects of focal damage on modular organization. Focal lesions are modelled as node and connection subtraction. Global hubs are nodes with strong connections between modules (also referred to as connector hubs). Damage to global hubs results in increases in network modularity. Local hubs are nodes with strong connections to other nodes within a module (also referred to as provincial hubs). Damage to local hubs results in decreased modularity.

To date, the most common approach adopted in simulation studies of focal lesions has involved developing a functional connectome (sometimes referred to as a “network”) based on human or animal data and then simulating lesions by systematically deleting nodes and/or connections from the network and, then, assessing the functional properties of the remaining network. The functional activity of the nodes are either simulated with mathematical models^17–24^ or based on real functional neuroimaging data (^25,26^; for a review see^15^). These studies have provided strong convergent evidence that: (a) focal lesions can have distant effects on the brain’s functional connectivity and that (b) this is especially true when the lesions affect brain regions that involve “hubs”, i.e., nodes that have widespread strong connections (functional or structural) to multiple regions. Furthermore, damage to different types of hubs has been shown to have distinctive effects on global network properties (Fig. 1)^18,23,27^.

Gratton et al.^2^ tested whether these simulation predictions were upheld in connectomes of resting-state fMRI data from a group of individuals with focal brain lesions (stroke, tumor and TBI). They confirmed the simulation predictions that focal damage can result in far-reaching disruption of the brain’s functional modular structure, even in the intact hemisphere. Furthermore, they showed that the extent of this disruption was related to the connectivity properties of the nodes that were affected. In other words, the nature of the disruption was determined by the extent to which focal damage primarily affected nodes that connect across multiple modules or mainly within one module (these two are often referred to, respectively, as *connector* and *provincial hubs,* and here we will refer to them as *global* and *local hubs*, see Fig. 1). Gratton et al.^2^ found that, as predicted, lesions that primarily affected global hubs had greater impact on overall functional modularity than did lesions that primarily affected local hubs. However, they did not find the predicted directionality of the relationships between global and local hub damage and modularity (Fig. 1): Simulation studies had predicted that greater damage to global hubs should increase modularity as damage to global hubs reduces communication/integration across the network, thus increasing module segregation and overall network modularity (e.g.^18,23,27^). However, Gratton et al.^2^ found the reverse relationship in real lesions such that greater global hub damage resulted in lower modularity (less overall segregation). With regard to local hub damage, the prior simulation work predicted that greater damage to local hubs should decrease modularity (Fig. 1) since damage to local hubs reduces communication/integration within the affected module, thus reducing overall network modularity. However, Gratton et al.^2^ found no significant relationship between the magnitude of local hub damage and overall modularity in real lesions. The focus of the Gratton et al. conclusions was on their confirmation that real lesions to global hubs can cause widespread disruption of the brain’s functional modular structure. Nonetheless, they did note that the discrepancies between their results and the simulation predictions with regard to the directionality of effects implied that “the loss of global hubs does not simply cause the loss of a select number of edges, but rather leads to a re-organization of the graph… (^2^ p. 1280)”.

**Figure 2:**
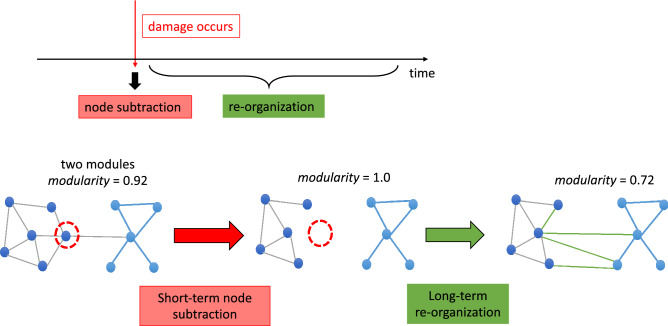
Schematic illustration of the hypothesis under consideration. The effects of focal lesion consist of two phases: *short-term node subtraction* and *long-term re-organization* that unfolds over time. In the specific example depicted, following global hub subtraction, network modularity increases due to reduced communication between modules. During re-organization, modularity decreases due to connectivity changes between the remaining nodes (modularity was quantified by *Newman’s Q*).

In the current investigation, we followed-up on the discrepancy between predicted and observed functional connectome properties that Gratton et al.^2^ reported. We hypothesized that the effects of focal lesions consist of two phases (or components): *short-term node subtraction* and *long-term functional re-organization* (Fig. 2). Subtraction involves the removal of lesioned nodes and connections, while long-term re-organization involves additional dynamic changes that might occur throughout the brain over time in response to the subtraction. With few exceptions, the modelling of focal lesions has been limited to the subtraction phase (but see^20^) in which lesions have been simulated as the subtraction of network nodes and connections. According to our hypothesis, the reason that the Gratton et al.^2^ findings were not consistent with previous simulation studies is because at least some of the network properties observed following real lesions do not arise from the subtraction phase but, instead, from the long-term re-organization phase (Fig. 2). As stated in a review by Aerts et al.^15^ “… mere removal of nodes and their links – used to simulate random brain lesions – does not capture the complexity of how disease processes affect the connectome” (^15^ p. 3079).

To evaluate the node subtraction + re-organization hypothesis, we analyzed the functional connectomes of a group of individuals with chronic stroke (N = 25) who suffered from written and spoken language impairments as well as from age-matched healthy controls (N = 10 and a second validation group N = 13). We used “background connectivity” to compute the functional connectomes. This approach uses the time-course from task-based fMRI after accounting for task-related activation (e.g.^28,29^; also see a similar approach in^30–33^. See “Functional connectivity estimation”). Various studies have shown that background connectivity is qualitatively similar to resting-state functional connectivity (FC) with subtle task-related modulations^31–34^. Therefore, we assume that the task-based FC we report in this work largely resembles the FC observed at rest, and additionally may be more likely than resting-state FC to reflect features associated with the language deficit/s shared by the stroke participants. Note, however, that it is not a goal of the current investigation to evaluate a specific characterization of task-based FC. Using these functional connectomes, we evaluated the same network measures examined by Gratton et al.^2^, i.e., *modularity* (note that we used the italicized *modularity* to refer to the specific graph-theoretic measure, *Newman’s Q*^35^; unitalicized use of the term refers to the general concept of modularity), and two nodal measures PC and WD. The latter two, PC (*participation coefficient*) and WD (*within-module degree z-score*) serve to measure the extent to which nodes can be considered global and/or local hubs, respectively (^36^ see Fig. 1).

We carried out three analyses to evaluate the hypothesis that the effects of focal lesion on functional connectomes are not limited to the effects of short-term node subtraction but instead involve long-term functional re-organization (Fig. 2). The goal of Analysis 1 was to replicate prior computer simulation studies (e.g.^18^) that examined the effects of targeted lesions on functional modularity*.* A key difference from the previous work is that we used human connectome data rather than mathematically based or data from animal studies. However, similar to previous studies we simulated *targeted lesions* to specifically examine the roles of global or local hubs (Fig. 1) by selectively removing these hubs within the connectomes of healthy individuals. Analysis 2 was the central analysis of this work as its goal was to evaluate our hypothesis that the effects of real lesions cannot be reduced to node subtraction. To do so, we simulated the effects of node subtraction by creating *pseudo-lesions* and then compared their network properties with those of the *real lesion* connectomes. Pseudo-lesions were created using healthy control connectomes from which we removed the specific nodes (and their connections) that were lesioned in the real stroke participants. We assumed that pseudo-lesioned connectomes should have the network properties predicted by node subtraction only. On this basis, we predicted that if node subtraction is *not* an adequate explanation of the effects of focal lesions, then the network characteristics of real-lesion connectomes should be inconsistent with the network properties of the pseudo-lesioned ones (as in Gratton et al.^2^). In other words, we assumed that discrepancies between the real-lesioned and the pseudo-lesioned connectomes primarily reflect the effects of the long-term re-organization phase of the brain’s response to focal lesions. In Analysis 3, to further substantiate the notion of functional network re-organization in real lesions, we evaluated whether and how the neurotopographical distribution of nodes that acted as global and local hubs differed in the real and pseudo-lesions. This analysis served to characterize the types of specific network changes that go beyond what is observed following short-term node subtraction and, thus, reflect long-term re-organization of real functional connectomes subsequent to focal lesions. The procedure of generating the different types of lesioned connecotomes is depicted in Fig. 4.

## Methods

### Participants

Twenty-five individuals (nine females) who had suffered a single left hemisphere stroke (> 1 year post stroke, mean 79 months, SD = 57) participated in the study. The mean age of the participants with lesions (referred to as the *Real-Lesion Group*) at the time of data acquisition was 61.6 (SD = 10) and years of education from 12 to 19. Before the stroke, all but three participants self-reported being right-handed as assessed by the Edinburgh Handedness Inventory^37^. The distribution of the lesions followed a typical lesion pattern of middle cerebral artery (MCA) strokes that involved the greatest density of damage in the left insula (Supp Fig. 1). Two groups of healthy controls were scanned with the same protocol as the participants with lesions. The first group (*HC Group*) consisted of 10 age- and education-matched, neurologically healthy individuals (eight females, mean age = 60.7, SD = 12, years of education: 12–18 years, all were right-handed). The second healthy control group was used as the validation dataset (*HC*^*v*^* Group,* reported in Supplementary Information Sect. 3), which consisted of 13 participants (ten females, age: 57 ± 7.39, with 12–18 years of education, three were left-handed). All participants provided informed consent following procedures approved by Johns Hopkins University Institutional Review Board.

### Cognitive/language assessment

The lesioned participants were recruited for a study on post-stroke written language production deficits and they also exhibited other language and motor deficits. The participants were administered an extensive battery of cognitive and language tests. Prior reports on subsets of these individuals include^13,38,39^.

### Functional magnetic resonance imaging (fMRI) data acquisition

All participants were scanned with multiple structural and functional MRI scanning protocols, including a spelling task and T1-weighted structural imaging and, additionally, a T2-weighted fluid-attenuated inversion recovery MRI (FLAIR) was obtained for the stroke participants. While scanning was carried out both before and after a 12-week dysgraphia rehabilitation period, this investigation evaluated only pre-treatment data. Each participant was scanned for four runs (7.7-min each) of a spelling task (see Sect. 1 in “Supplementary Information” for details) distributed over two scanning sessions for the Real Lesion Group and one session for the HC Groups, generating a total of 30.8 min of data for the functional connectivity (FC) analysis.

All MRI data were collected using a Phillips 3T scanner at the F.M. Kirby Research Center for Functional Brain Imaging (Baltimore, MD). The acquisition parameters for all participant groups were as follows: TR = 1500 ms, TE = 30 ms, FOV = 216 × 120 × 240 mm (ap, fh, rl), flip angle = 65°, voxel dimension = 1.875 × 1.875 × 3 mm (1.5 mm interslice gap), data matrix = 128 × 128 × 27. One run contained 308 TRs (7.7 min). The T1-weighted structural MRI acquisition parameters were as follows: TR = 6 ms, TE = 2.91 ms, FOV = 256 × 256 × 176 mm (ap, fh, rl), flip angle = 9°, voxel dimension = 1 × 1 × 1 mm, data matrix = 256 × 256 × 176. The acquisition parameters of the stroke participants’ FLAIR images were TR/TE/TI = 9000/90/2500 ms, FOV = 220 × 175 × 220 mm (ap, fh, rl), voxel dimension = 0.86 × 0.86 × 5 mm (no interslice gap), data matrix = 256 × 228 × 35.

### MRI data analysis

#### Preprocessing

FMRI data preprocessing and functional connectivity estimation (see “Functional connectivity estimation”) were identical to our previous study^13^. Functional data were preprocessed with FEAT in FSL 5.0.9^40^ with the following pre-processing steps: removal of the first 4 volumes of each run, motion correction with MCFLIRT, slice-timing correction, non-brain tissue removal with BET, spatial smoothing using a 5 mm FWHM Gaussian kernel, grand-mean intensity normalization, high-pass temporal filtering (0.01 Hz). Registration was carried out with FSL’s two-step method: functional images were first registered to the subject’s T1 image with the boundary-based registration (BBR) method, then to standard MNI space (spatial resolution 2 mm). A lesion mask for each participant was manually drawn by a trained image analyst on the original T1 image with MRIcron (http://people.cas.sc.edu/rorden/mricron/index.html). The masks were then aligned to standard MNI space using the transformation calculated with the T1 image. The lesion distribution map is shown in Supp. Fig. 1).

#### Functional connectivity estimation

First, a standard general linear model (GLM) analysis was carried out with FEAT in FSL^40^ and all task-related regressors were convolved with a double-gamma hemodynamic response function provided in FSL. To account for task-related co-activation, we then estimated functional connectivity (FC) using the residuals of the GLMs, an approach that has been referred to as “background connectivity” (e.g.^28^) and is often used in task-based FC studies (e.g.^31,32^). The Python package *Nilearn* was used for FC estimation and visualization (http://nilearn.github.io). We used the 264 locations identified by Power et al.^41^, excluding 29 locations in subcortical areas based on the segmentation template provided in FSL (*MNI152_T1_2mm_strucseg.nii*). For the remaining 235 locations, we extracted the averaged time-series from 5 mm-radius spheres centered at each location. Prior to calculating pairwise-connectivity, we additionally regressed out the first derivatives of the six motion parameters and the averaged time-series of the brainstem, the cerebellum, the basal ganglia, and the ventricles defined by the segmentation template in FSL. For each run (7.7 min), we calculated pairwise Pearson correlations (with Fisher’s z-transform) for the 235 nodes (and fewer for each Real-Lesion Group participant, see below). For each participant, the correlation matrices of the four runs were averaged to yield one connectivity matrix per participant.

#### Identifying the reference modular organization

**Figure 3:**
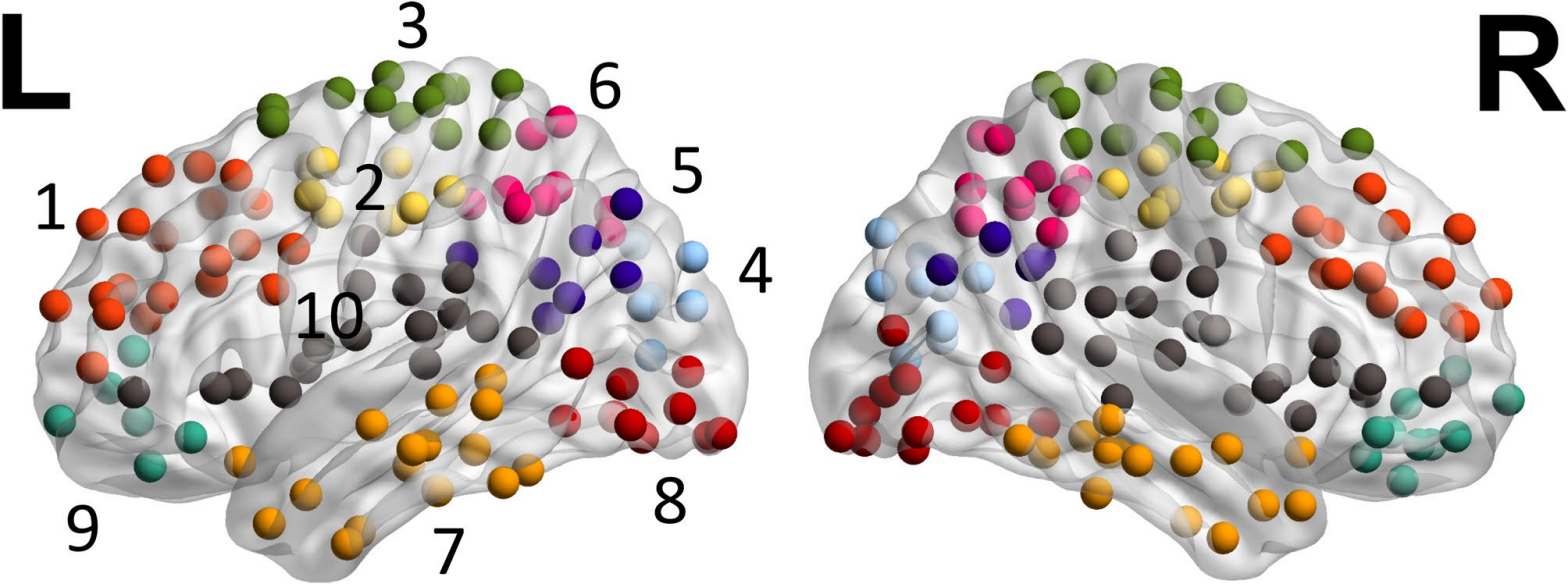
The reference modular organization. Using the Healthy Control Group connectomes (HC, N = 10), a 10-cluster clustering solution was calculated via hierarchical clustering analysis. The color-coding scheme and the neuroanatomical labels of each cluster are described in “Identifying the reference modular organization”. The figure was generated with BrainNet Viewer.

We identified a “reference” modular organization which was used in calculating the graph-theoretic properties that were evaluated in subsequent analyses. This modular organization was reported previously in^13^. Briefly, it was derived by applying hierarchical clustering (agglomerative) with Ward's criterion implemented in SciPy (https://scipy.org) to the averaged correlation matrix of the HC Group. We used a 10-cluster clustering solution (Fig. 3) and the ten clusters were assigned the following neuroanatomical labels: (1) prefrontal, (2) ventral frontoparietal, (3) dorsal frontoparietal, (4) medial occipital, (5) occipitoparietal, (6) posterior parietal, (7) temporal, (8) ventral occipitotemporal (VOT), (9) ventromedial prefrontal, and (10) perisylvian.

#### Calculating graph-theoretic measures and identifying reference hubs

All graph-theoretic analyses were carried out with the *brain connectivity toolbox*^42^. A proportional threshold was first applied to each connectivity matrix to preserve the top connections and then the thresholded connectivity matrix was binarized to create an undirected binary graph. A set of commonly used proportional threshold values were applied (e.g.^2,12^), i.e., 10%, 20%, 25%, 30%, 40%, and to maximize stability, the results reported here were obtained by averaging across the thresholds (except when examining specific hub distributions, see “Analysis 3: The neurotopography of global and local hubs in pseudo and real lesions”).

First, whole-brain *modularity* (*Newman’s Q*^35^) based on the reference modular organization (as described in Sect. Identifying the reference modular organization, Fig. 3) was calculated for each participant in the HC and Real-Lesion groups, as well as for the different kinds of artificial lesions (see “Analysis 1: Replication of simulated targeted-lesions” and “Analysis 2: Comparison of pseudo and real lesions: modularity, PC and WD” for the artificial lesion calculations). In addition, we also calculated *modularity* per hemisphere using only within-hemisphere connections (note that all ten clusters were bilateral). Second, we computed the two nodal measures, *participation coefficient* (PC) and *within-module degree z-score* (WD)^36^ for each node in each matrix. Third, based on the PC and WD measures from the HC Group, we identified “reference” global and local hubs. To do so, we first averaged the PC and WD values for each node across the HC Group to generate PC and WD whole-brain templates. Then, for the two templates, we identified the global and local hubs as those nodes with PC or WD values that were greater than one standard deviation above the mean of all the nodes in the respective templates.

#### Analysis 1: Replication of simulated targeted-lesions

**Figure 4:**
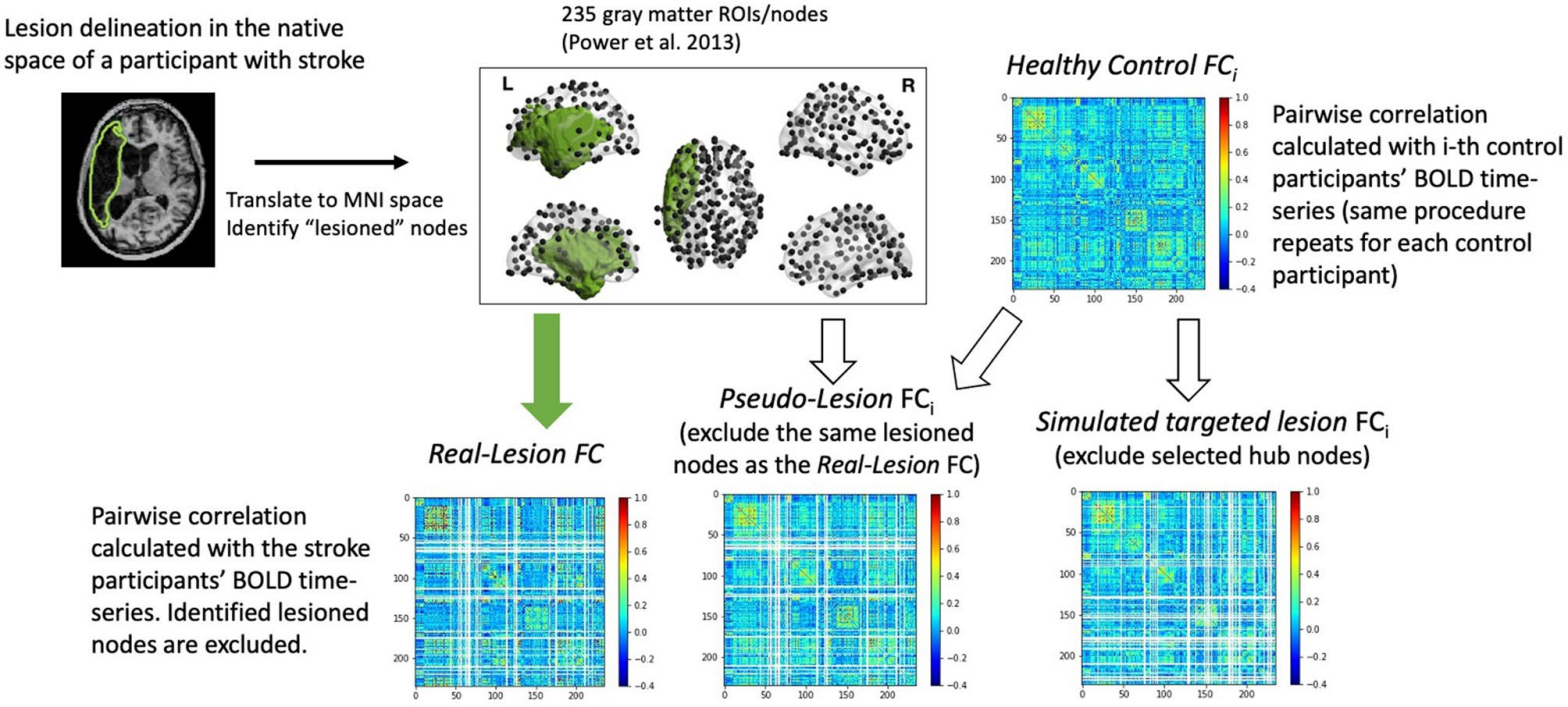
Procedures for generating Real-Lesion, Pseudo-Lesion, and Simulated Targeted-Lesion functional connectomes (FC). For each participant, their functional connectome was calculated based on 235 gray matter ROIs (10 mm-radius spheres), resulting in a 235-by-235 pairwise Pearson correlation matrix (see “Functional connectivity estimation”). **Real-Lesion FC** (bottom-left): for each participant, a lesion mask was first manually drawn on the structural image, then the lesion mask was co-registered to the MNI standard space to identify the “lesioned nodes” (see “Preprocessing” and “Analysis 2: Comparison of pseudo and real lesions: modularity, PC and WD”) and, the correlation values of the lesioned nodes (rows and columns of the matrix, marked as blank in the figure) were excluded from subsequent analyses. **Pseudo-Lesion FC** (bottom center): for each healthy control’s connectome, correlation values corresponding to the “lesioned nodes” were excluded from subsequent analyses. The FC from one healthy control is depicted in which the same rows and columns as in the Real-Lesion FC are left blank. **Simulated Targeted-Lesion FC** (bottom right): for each healthy control’s connectome, correlation values of the identified global/local hub nodes (Fig. 8a) were excluded from subsequent analyses.

We simulated previously reported “targeted attacks” to evaluate if these would yield the same effects^18,26,27^ using our data set of healthy functional connectomes. Specifically, we examined if, as reported in this earlier work, removing the global nodes would increase system segregation (i.e., higher *modularity*) while removing local hubs would have the opposite effect (Fig. 1). We used the reference global and local hubs identified as described above in “Calculating graph-theoretic measures and identifying reference hubs” (see the identified hubs in “Analysis 3: Functional re-organization after brain damage involves changes in hub distribution: “Lost” and “new” global and local hubs” and Fig. 8a), and we removed those targeted hub nodes and their connections from each HC participant’s connectivity matrices (i.e., functional connectomes) to create targeted global and local hub attacks, respectively. We refer to these as “*simulated targeted lesions*” (Fig. 4). *Modularity* values were then computed for each *Simulated Targeted-Lesion* connectome using the same methods as described in “Calculating graph-theoretic measures and identifying reference hubs”, and then compared these values to *modularity* calculated from the intact healthy connectomes (HC group) with paired t-tests.

#### Analysis 2: Comparison of pseudo- and real-lesions: *modularity*, PC and WD

The procedure for generating the lesioned connectomes is illustrated in Fig. 4. We first identified nodes (i.e., 5mm-radius spheres) that had more than 25% voxels damaged according to the lesion delineated on the structural scans of each of the 25 participants in the Real-Lesion Group. We considered these to be “lesioned nodes” and each set of these nodes constituted a lesion mask. For each of the 25 participants with lesions (the Real-Lesion Group), those lesioned nodes and all their connections were removed from the connectome. After applying the lesion masks, the number of remaining nodes ranged from 184 to 232. Then, to generate the “pseudo-lesioned” connectomes, we removed the lesioned nodes/connections of each of the 25 lesion masks from each of the connectivity matrices (connectomes) of the ten individuals in the HC Group, resulting in a total of 25 × 10 *pseudo-lesion* connectomes (referred to as the Pseudo-Lesion Group hereafter). Note that, unlike the simulated targeted lesions where the neurotopography of the artificial lesions was based on the distribution of nodal attributes (PC and WD), for the pseudo-lesions the damage was obtained from real stroke participants and concentrated in the middle cerebral artery (MCA) territory (Supp Fig. 1), the most commonly damaged area in stroke. Using the Real- and Pseudo-Lesion as well as Healthy Control (non-lesioned) connectomes the network analyses proceeded as follows.

First, *modularity* values were computed for each Healthy Control (n = 10), Real-Lesion (n = 25) and Pseudo-Lesion connectome (n = 250, 25-lesion-mask by 10-HC) with the same methods as described in “Calculating graph-theoretic measures and identifying reference hubs”. Differences across the three groups were examined with independent t-tests.

Second, for each lesion mask, we calculated two damage scores to quantify the extent of damage to the global and local hubs respectively, following the approach used in^2^. Specifically, using the PC and WD whole-brain templates derived from the HC Group (described in “Calculating graph-theoretic measures and identifying reference hubs”), for each lesion mask, we averaged the PC or WD values of the damaged nodes to obtain a *PC/WD damage* score for each lesion mask. For example, if a lesion mask included nodes with high PC values in the PC template derived from the healthy control group, this lesion mask would have a high PC damage score, indicating greater extent of global hub damage. Then the relationships between PC/WD damage scores and *modularity* values across the 25 lesion masks were characterized using Pearson correlation for both the Real- and the Pseudo-Lesion Groups. Specifically, for the Pseudo-Lesion Group which consisted of 25-lesion-mask by 10-HC *modularity* values, we calculated the correlation between the 25 *modularity* values derived from the 25 lesions and the 25 PC/WD damage scores separately for each of the 10 HC participants, resulting in ten correlation values. Significance of the correlation values for each group was evaluated using 10,000 permutation testing.

In addition, the difference in correlation values between the Real-Lesion and the Pseudo-Lesion Group was assessed with a bootstrapping resampling method: ten correlation values from the Pseudo-Lesion Group were randomly selected (with replacement) and the bootstrap group mean was computed; the procedure was repeated 10,000 times, yielding a bootstrap distribution of 10,000 correlation values. Then, the Real-Lesion Group’s correlation value was compared against the bootstrapped distribution of correlation values to obtain the p-value.

We also repeated the same analyses for each hemisphere using hemispheric *modularity,* i.e., *modularity* calculated for each hemisphere considering only within-hemisphere connections. Note that analyses of the right-hemisphere (RH) for the Pseudo-Lesion group are omitted because the lesions were limited to the left hemisphere and hence there were no nodes or connections removed from the right hemisphere Pseudo-Lesion connectivity matrices. In other words, for the pseudo-lesion connectomes, since there is no “node subtraction” in the right hemisphere, the RH connectomes for the Pseudo-Lesion Group are identical to those of the Healthy Control Group, and hence results of RH *modularity* for the Pseudo-Lesion group are not reported.

We also examined different thresholds for identifying the “lesioned nodes”. As mentioned above, in the main analyses, we identified nodes with more than 25% of their voxels damaged as “lesioned”. We also examined different values of this threshold (i.e., 10%, 20%, 30%) and report the results in Supplementary Information 4. The results were highly consistent across the different values examined.

#### Analysis 3: The neurotopography of global and local hubs in pseudo and real lesions

To better understand the mechanisms of re-organization, we identified global and local hubs in the Real- and Pseudo-Lesion Groups using the same method as was used to identify the reference hubs in the undamaged HC connectomes (i.e., nodes with nodal hub measures (PC or WD) greater than one standard deviation above the mean. See “Calculating graph-theoretic measures and identifying reference hubs”). Then we directly compared the topographical distributions of the global and local hubs of the Pseudo- and the Real-Lesion groups to identify “lost” hubs (i.e., local or global hubs present in the Pseudo-Lesion Group but not in the Real-Lesion Group) and “new” hubs (i.e., local or global hubs present in the Real-Lesion Group but not in Pseudo-Lesion Group).

### Evaluation of white matter hyperintensities (WMH)

The functional connectomes were constructed based on gray matter nodes only (see “Functional connectivity estimation”) and, hence, only the consequences of gray matter damage were evaluated. However, gray matter signals could potentially be indirectly affected by other factors, including white matter damage and other pathological changes after stroke (see the “General discussion” for further discussion of this topic). White matter hyperintensities (WMH) are one factor that has received considerable attention in the literature, although their impact on functional connectivity has not been established and is still under investigation (see review by^43^). To examine this possibility, for each participant in the Real-Lesion Group we evaluated their T2-weighted fluid-attenuated inversion recovery MRI (FLAIR) scans for the presence of white-matter hyperintensities (WHMs). We visually inspected the FLAIR scans following the Fazekas guidelines^44^. Given that evaluation of tissue properties in the ipsi-lesional hemisphere can be complicated by the lesion, we focused on the contra-lesional hemisphere to identify WMHs present in the periventricular spaces and/or the deep white matter. Each WMH (periventricular or deep white matter) was rated using the 0–4 Fazekas scale. Then for the participants for whom both periventricular and deep white matter ratings were below 2, we categorized the participant' as absent/mild WMH, and those with either rating equal or above 2 were categorized as moderate/severe WMH. To examine whether the presence of WMHs had an effect on the functional connectivity properties examined in this study, we compared the two groups (absent/mild vs. moderate/severe) in terms of *modularity* as well as age, since WMHs have been most often associated with aging*.*

## Results

### Analysis 1: Simulated targeted attacks result in functional network changes consistent with simulation predictions

**Figure 5:**
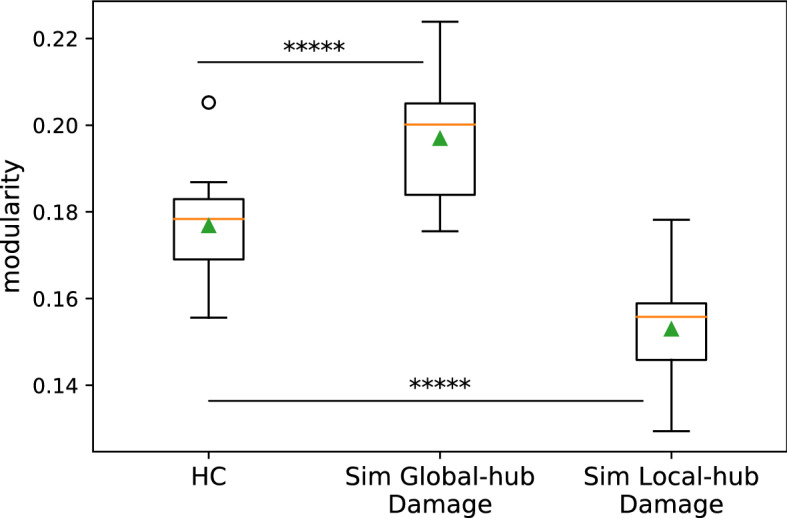
*Modularity* resulting from simulated targeted lesions. *Modularity* values of the Healthy Control Group (HC, N = 10) compared (via paired t-tests) to *modularity* values resulting from the two types of targeted attacks (removal of global or /local hubs from healthy control connectomes). Left to right: HC, simulated global hub lesions, simulated local hub lesions. As predicted by the model (Fig. 1), damage to global hubs caused an increase in *modularity*, whereas damage to the local hubs caused a *modularity* decrease. *****p < 10e − 06.

Analysis showed that the simulated lesions targeting either the global or local hubs caused *modularity* to change in the predicted directions (Fig. 1). As shown in Fig. 5, removing the global hubs from the HC Group’s connectomes led to higher *modularity* (paired t-test t(9) = 10.58, Bonferroni p = 4.52e − 06), while, in contrast, removing the local hubs resulted in lower *modularity* (t(9) = − 25.14, Bonferroni p = 2.4e − 09). The results matched the model and simulation predictions (Fig. 1) such that by removing global hubs and their *inter-module* connections, the modules become more segregated, increasing overall brain *modularity*. Conversely, removing local hubs and their *intra-module* connections degrade the individual modules, reducing overall brain *modularity*.

### Analysis 2: Comparisons between pseudo- and real-lesions *modularity*, PC and WD

#### *Modularity* in real and pseudo-lesions

**Figure 6:**
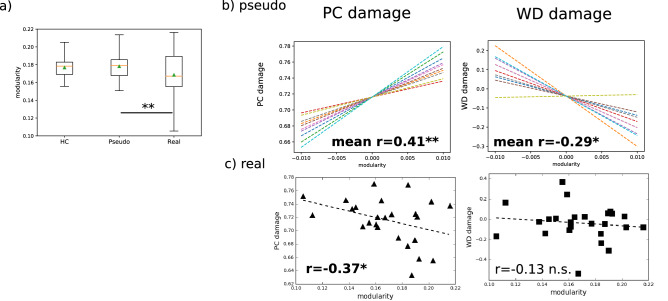
Whole brain *modularity* and global (PC) and local hub (WD) damage in healthy controls (HC), Pseudo- and Real-Lesions. (**a**) *Modularity* of the Healthy Control (HC, N = 10), Pseudo-Lesion (N = 250), and Real-Lesion Groups (N = 25). Neither Pseudo- nor Real-Lesion Groups differ from the HC Group, but the Real-Lesion Group shows lower *modularity* than the Pseudo-Lesion Group. (**b**) Relationship between *modularity* and the magnitude of PC (left) and WD damage (right) for the Pseudo-Lesion Group. In both panels, each of the ten lines corresponds to the correlation between the PC/WD damage of the 25 lesion masks and the resulting *modularity* values obtained after applying the lesion masks to one (of  the 10) HC participant’s functional connectome (note that the plotted *modularity* values here were centered to zero for visualization purposes). For the Pseudo-Lesion Group, PC damage scores are positively correlated with *modularity* and that the relationship for WD damage is negative, consistent with model predictions (Fig. 1). (**c**) Relationship between *modularity* and magnitude of PC (left, triangles) and WD damage (right, squares) for the Real-Lesion Group. In contrast to the Pseudo-Lesion Group shown in (**b**), PC damage scores for the Real-Lesion Group are negatively correlated with *modularity*, while WD damage and *modularity* are not correlated. *p < 0.05, **p < 0.01.

We compared *modularity* values for the three groups (HC, Real-Lesion and Pseudo-Lesion). As shown in Fig. 6a, neither the Real- nor the Pseudo-Lesion Group differed from the HC group (HC vs. Pseudo: t(258) = − 0.31, HC vs. Real: t(33) = 0.91, both p > 0.1 with Bonferroni correction), although Real-Lesion Group *modularity* was significantly lower than for the Pseudo-Lesion Group (t(273) = 2.97, Bonferroni p = 0.01).

We also evaluated *modularity* for each hemisphere by considering only the within-hemisphere connections. In the ipsi-lesional hemisphere (LH), we found no differences across the three groups (HC vs. Pseudo: t(258) = − 0.88, HC vs. Real: t(33) = − 1.01, Pseudo vs. Real: t(273) = − 0.74, all p > 0.1, Bonferroni corrected). For the contra-lesional hemisphere (RH), as explained in “Analysis 2: Comparison of pseudo and real lesions: modularity, PC and WD”, because there were no lesions in the RH from which to generate Pseudo-Lesions, we only compared the HC and the Real-Lesion Group. Similar to what was observed for the whole-brain and the LH, the two groups did not differ (t(33) = 1.01, p = 0.32).

Finally, for the Real-Lesion Group, *modularity* did not differ between males and females (whole-brain: t(23) = 1.21, p = 0.24; LH: t(23) = 0.8, p = 0.43, RH t(23) = 1.39, p = 0.18) and did not correlate with lesion volume (whole-brain: r = 0.03, p = 0.44; LH: r = 0.16, p = 0.2; RH: r = − 0.004, p = 0.5).

#### Different relationships between PC or WD damage and *modularity* in real compared to pseudo-lesions

To examine the relationship between hub damage and modular organization in real and pseudo-lesions, we computed correlations between the PC and WD damage scores (i.e. extent of global and local hub damage respectively) and *modularity* values for the Real and Pseudo-Lesion Groups for the whole brain as well as for each hemisphere. We found that for the Pseudo-Lesion Group, global hub damage scores (PC damage) were positively correlated with *modularity* (mean r = 0.41, p = 0.0038, Fig. 6b) as predicted by the model, prior simulation studies and the results of Analysis 1, such that as more global hubs were damaged, system segregation increased (Fig. 1). In contrast, for the Real-Lesion Group, *modularity* was negatively correlated with the degree of PC damage (r = − 0.37, p = 0.038, Fig. 6c), contradicting model predictions (difference between Real- and Pseudo-Lesion correlations: p < 0.0001 by 10,000 bootstrapping test). With regard to the relationship between WD damage and *modularity*, for the Pseudo-Lesion Group, the WD damage was negatively correlated with *modularity* (r = − 0.29, p = 0.0352, Fig. 6b), again consistent with the model predictions that removing local hubs will produce deterioration of the targeted modules and result in a decrease in overall *modularity* (Fig. 1). However, for the Real-Lesion Group, no significant correlation was found between WD damage and *modularity* (r = − 0.13, p = 0.28, Fig. 6c), which was significantly different from the Pseudo-Lesion group (p = 0.0003 by 10,000 bootstrapping test).

**Figure 7:**
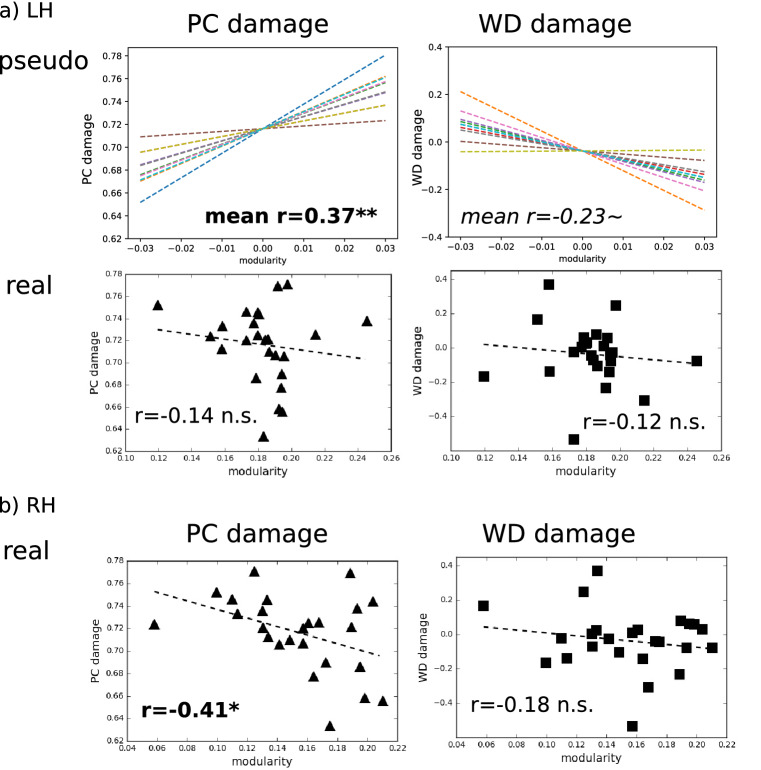
Relationship between hemispheric *modularity* and hub damage. (**a**) Relationship between left hemisphere (LH, ipsi-lesional) *modularity* and PC (left) and WD damage (right) for the Pseudo-Lesion (top) and the Real-Lesion (bottom) Groups. Similar to the whole-brain results (Fig. 6), the two groups show different relationships between *modularity* and hub damage. (**b**) Relationship between right hemisphere (RH, contra-lesional) *modularity* and PC (left, triangles) and WD damage (right, squares) for the Real-Lesion Group. Note that because there are no damaged RH nodes, for the Pseudo-Lesion Group (see “Analysis 2: Comparison of pseudo and real lesions: *modularity*, PC and WD”), this analysis is omitted. *: p < 0.05, **: p < 0.01, ~ : p < 0.1.

Similar patterns were observed for the relationships between PC and WD damage scores and *modularity* in the ipsi-lesional hemisphere (LH, Fig. 7a, left). First, for the Pseudo-Lesion Group, PC damage was positively correlated with *modularity* (r = 0.37, p = 0.0049), while for the Real-Lesion Group the correlation was negative (r = − 0.14, p = 0.26). Although the latter result did not reach significance, it differed significantly from the correlations of the Pseudo-Lesion Group (p < 0.0001). For WD damage (Fig. 7a, right), in the left hemisphere, similar to the whole brain, the Pseudo-Lesion Group exhibited a marginally significant negative correlation with *modularity* (r = − 0.23, p = 0.07), whereas the Real-Lesion Group did not (r = − 0.12, p = 0.28). Importantly the correlations of the two groups were also significantly different from one another (p = 0.0023).

Regarding RH (contra-lesional) *modularity*, for the Real-Lesion Group, we found the same results as observed in the whole brain such that PC damage was negatively correlated with *modularity* (r = − 0.41, p = 0.02) and WD damage was not correlated with *modularity* (r = − 0.18, p = 0.20, Fig. 7b). Note that as the Pseudo-Lesion Group did not have nodes and connections removed from the RH, the RH analysis was not possible for the Pseudo-Lesion Group.

In summary, although effects of the simulated targeted lesions (“Analysis 1: Simulated targeted attacks result in functional network changes consistent with simulation predictions”) and the relationships between PC, WD and *modularity* in pseudo-lesions were as predicted by the model (Fig. 1), the predictions were not confirmed in individuals with real lesions even though the same sets of nodes and connections were removed from the functional connectomes in both Real- and Pseudo-Lesion Group. This discrepancy between real and pseudo-lesions indicates that real lesions result in connectivity changes that can not be fully accounted for by simple node subtraction, signaling the presence of long-term re-organization, examined in the next analysis.

### Analysis 3: Functional re-organization after brain damage involves changes in hub distribution: “Lost” and “new” global and local hubs

**Figure 8:**
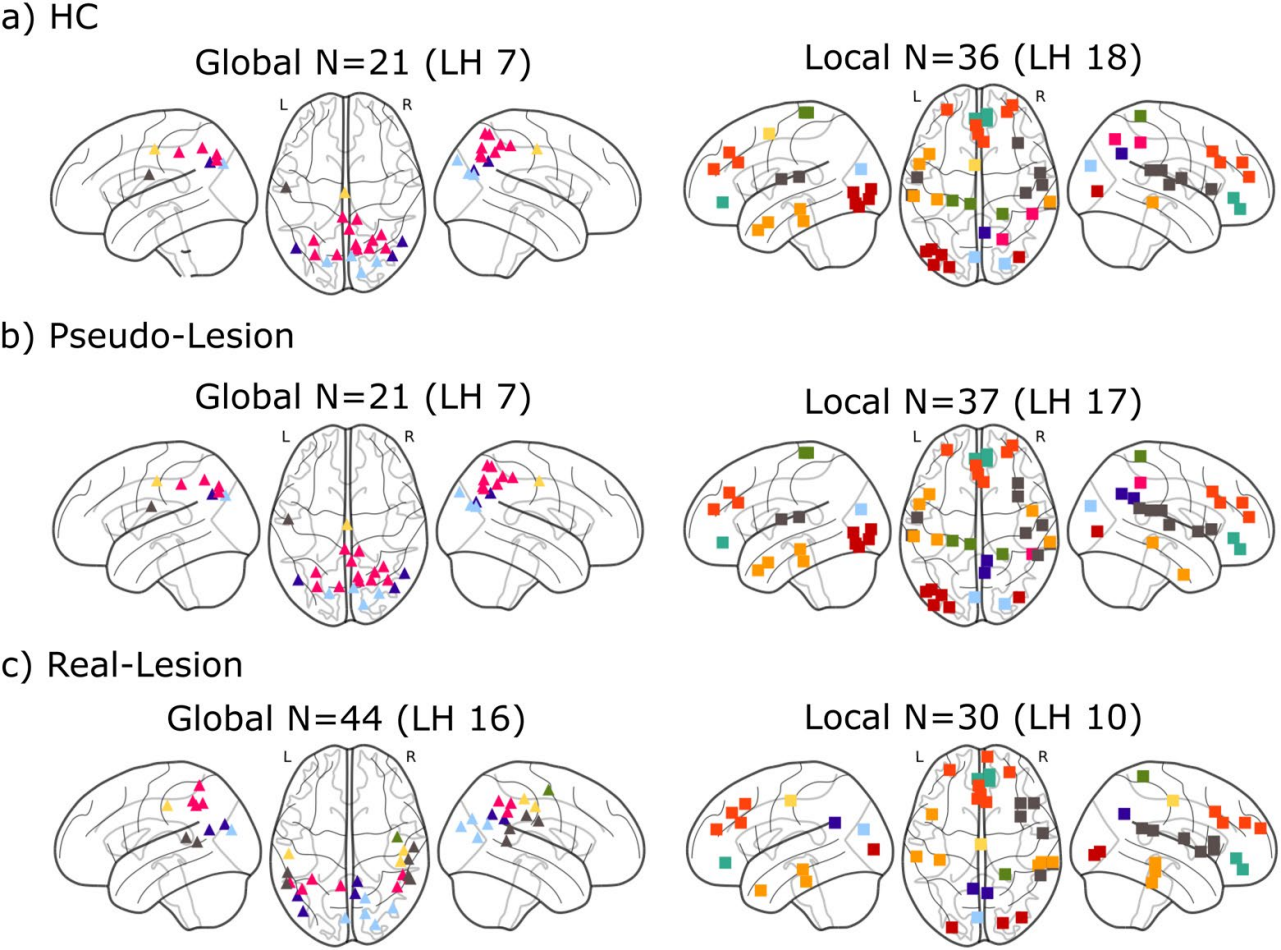
Global hubs (triangles, left) and local hubs (squares, right) identified for each group. (**a**–**c**) Healthy Controls (HC), Pseudo-Lesions, Real-Lesions. The colors indicate module membership as in Fig. 3 (“Identifying the reference modular organization”). The hubs identified for the HC Group (**a**) are also referred to as reference hubs. The number of left hemisphere (LH) hubs are indicated in parentheses. The visualization was created with Nilearn (https://nilearn.github.io).

As described in “Calculating graph-theoretic measures and identifying reference hubs”, we identified the global and local hubs as the nodes with the top PC/WD scores, for each group. The results were consistent across the proportional thresholds, but for efficiency we present the results with a proportional threshold of 25%. With regard to global hubs, in both the HC Group and the Pseudo-Lesion Groups, these were concentrated in the bilateral parietal lobes (Fig. 8a,b, left); On the other hand, for the Real-Lesion Group a larger total number of nodes were identified as global hubs (44 compared to 21) and, furthermore, the distribution extended more anteriorly and inferiorly, especially in the RH (Fig. 8c, left panel, median values of the MNI coordinates: HC and Pseudo-Lesion [10, − 65, 42], Real-Lesion [11, − 57, 37]). In terms of local hubs, again the HC and the Pseudo-Lesion Groups had very similar distributions, with local hubs found in most modules and distributed bilaterally (Fig. 8a,b, right panel). In contrast, the local hubs in the Real-Lesion Group showed a rather different distribution such that, compared to both the HC and the Pseudo-Lesion Groups, the Real-Lesion Group “lost” hubs bilaterally and recruited new hubs either in the RH or around the midline, resulting in a more strongly right-lateralized distribution with 65% of local hubs in the RH for the Real-Lesion Group (chi-square = 3.33, p = 0.07), compared to 54% of the Pseudo-Lesion Group (chi-square = 0.243, p = 0.62, Fig. 8, right panel). Note that these differences between the Real-Lesion and Pseudo-Lesion groups cannot be explained by volume shifts due to the lesions because: (a) the fact that the Real-Lesion group had a greater number of global hubs indicates changes in functional connectivity rather than shifted locations and (b) spatial shift would not affect the RH and, for local hubs, the Real Lesion groups had multiple new hubs in the RH. To summarize, the Pseudo-Lesion Group showed very little change in the topographical distribution of global and local hubs relative to the Healthy Control Group while, conversely, the Real-Lesion Group exhibited a more extended distribution of the global hubs (although they were found in the same general area), and a shift of local hubs to the right hemisphere.

### Validation with a second healthy control cohort

We validated all of the findings reported in Analysis 1–3 with a second age-matched HC cohort (N = 13) whose data were not used in identifying the reference modular organization or the reference global or local hubs. We used this data set to generate new pseudo-lesions and to replicate the analyses reported in the previous sections (note that we refer to these groups as the HC^v^ and Pseudo^v^ Groups where “v” signifies “validation”). These analyses yielded the same results as observed with the first HC cohort. Details of the participants and results are reported in the Supplementary Information 3 (Supp Figs. 2, 3 and 4), here we summarize the main findings briefly.

First, regarding the simulated targeted lesions, as with the first HC cohort (Fig. 5), damage to the global hubs resulted in an increase in *modularity* (t(12) = − 10.0, p = 3.57e − 07), whereas simulated damage to the local hubs resulted in a decrease in *modularity* (t(12) = 17.59, p = 6.20e − 10). Second, there was no difference in whole brain *modularity* between HC^v^ and Pseudo^v^ (t(336) = − 0.52, p = 0.60) in the whole brain as well as in the ipsi-lesional hemisphere (i.e., LH: t(336) = 0.05, p = 0.976). Third, regarding the correlation between the PC/WD damage scores and *modularity*, we observed similar results as we reported with the first HC cohort, namely that *modularity* was positively correlated with PC damage (whole-brain: r = 0.37, p = 0.0049; LH: r = 0.34 p = 0.0111) and (marginally) negatively correlated with WD damage (whole-brain: r = − 0.20, p = 0.09; LH: r = − 0.20, p = 0.10). Finally, in terms of the hub distributions, the HC^v^ Group also showed very similar distributions of global and local hubs as was observed with the first HC cohort.

### Evaluation of white matter hyperintensity (WMH)

Given that the gray matter fMRI signals that were used to compute functional connectivity could have been affected by white-matter lesions, we also evaluated white matter hyperintensity (WMH) in the stroke participants (i.e., Real-Lesion Group, n = 25). We found seven participants with moderate to severe WMH, and 18 participants who showed no or only mild WMH. For these two subgroups, we did not find any differences in *modularity* (whole-brain: t(23) = − 0.67, p = 0.52; LH: t(23) = − 0.74, p = 0.47; RH: t(23) = − 1.09, p = 0.29) and the relationships between *modularity* and PC/WD damage reported in “Different relationships between PC or WD damage and modularity in real compared to pseudo-lesions” (Fig. 6c) were also seen in the 18 participants with absent/mild WMH after excluding the 7 participants with moderate/severe WMH  (*modularity*—PC damage: r = − 0.48, p = 0.02, *modularity*—WD damage: r = 0.11, p = 0.29). With regard to the relationship between WMHs and age, we found that the moderate/severe group was significantly older than the absent/mild group (t(23) = 2.91, p = 0.0078). This finding is consistent with the consensus that one of the most prominent risk factors for WMHs is age. However, we found no evidence that WMHs affect the functional connectivity measures that we evaluated in this study.

## Discussion

In this study, we investigated the effects of focal brain lesions on functional network properties by comparing real stroke lesions to artificial lesions that were created by removing nodes from the connectomes of healthy participants. We considered two types of artificial lesions: *simulated targeted lesions* that were designed to evaluate the effects of the targeted removal of specific node types (global or local hubs), and *pseudo-lesions* that corresponded to the removal of nodes that were lesioned in individuals with post-stroke language impairments (n = 25). While *simulated targeted lesion*s have been well investigated in computational neuroscience, to our knowledge, comparisons of real and pseudo-lesions have not been previously carried out (except by^12^ who did so for very different reasons than those pursued in this study). Our specific goal was to follow up on the Gratton et al.^2^ observation that certain network properties of connectomes of individuals with real focal lesions did not conform to key predictions of previous lesion simulation work as shown in Fig. 1. As discussed in the Introduction, we hypothesized that this discrepancy was due to the fact that previous simulations involved only node subtraction while, in the case of real lesions, node subtraction is followed by or occurs concomitantly with long-term network re-organization (Fig. 2). Given that pseudo-lesions involve simple node subtraction but not long-term re-organization, we predicted that the network properties of the connectomes with real lesions should differ from those with pseudo-lesions. Our findings clearly support these predictions and, while the results are perhaps not unexpected, they have important implications for future simulation studies. Furthermore, because the *pseudo-lesions* match with real lesions in node subtraction, the pseudo- vs. real-lesion comparison approach allows us to identify changes specifically due to long-term network re-organization, and has important implications for understanding post-stroke network re-organization.

### Evaluating the subtraction + re-organization hypothesis

Following Gratton et al.^2^, we focused on whole-brain functional modularity as measured by the graph-theoretic measure *modularity*, also known as *Newman’s Q*^35^, as well as the nodal properties of *participation coefficient* (PC) and *within-module degree z-score* (WD)^36^. Evaluating the subtraction + re-organization hypothesis involved three steps. (1) Using connectomes from healthy control participants, we replicated previous findings from lesion simulation^18,23,26,27^, see Fig. 1), namely that: (a) simulated lesions specifically targeting global hubs (high PC nodes) had the effect of reducing communication between modules, resulting in increased overall network *modularity* and (b) simulated lesions specifically targeting local hubs (high WD nodes) had the effect of collapsing individual modules, resulting in reduced overall network *modularity* (Fig. 5). This replication served to confirm that predicted changes resulting from simulating node subtraction (Fig. 1) were also seen in our functional connectivity dataset from healthy controls, which formed the basis of the subsequent analyses. (2) We evaluated whether the network properties observed in individuals with real post-stroke lesions could simply be explained by node subtraction by directly comparing the relationships between *modularity* and hub damage (global or local) in the real and pseudo-lesioned connectomes. When we did so, we found that the pseudo-lesioned connectomes showed the relationships predicted by the model, previous simulation research, and the results of Analysis 1 (Fig. 6b), whereas the opposite pattern was seen in the real-lesion connectomes (Fig. 6c) in which: (a) greater damage to the between-module, global hubs (PC damage) was associated with *lower modularity* and (b) no systematic association was found between *modularity* and damage to the within-module, local hubs (WD damage). These discrepancies between the real and pseudo-lesioned connectome properties directly support the claim that the consequences of focal lesions involve more than node subtraction and imply long-term network re-organization. (3) If, as we hypothesized, the findings in (2) correctly indicate that long-term functional re-organization has taken place subsequent to real lesions, then we expect significant changes in nodal roles within the remaining functional connectome. Consistent with this prediction, we found that the neurotopographic distribution of global and local hubs differed between the real and pseudo-lesioned connectomes (Fig. 8).

### Focal lesions and network re-organization

Node subtraction corresponds to the deletion of the nodes and their functional connections, with no changes in the functional coupling among the remaining nodes (Fig. 2). However, if re-organization is a dynamic long-term process that allows for the creation/strengthening and the weakening/removal of connections in the remaining network, then more radical changes in network organization would be expected following focal lesions. To evaluate this possibility, we examined the topographical distribution of the global and local hubs in real-lesion connectomes compared to those of the pseudo-lesioned connectome (which instantiated only static node subtraction). As shown in Fig. 8, we found that the real and pseudo-lesioned connectomes differed considerably in the distribution of both local and global hubs. While the pseudo-lesioned connectomes (implementing node subtraction) scarcely differed from the non-lesioned healthy connectomes in terms of the number and distribution of local and global hubs, the real lesion connectomes exhibited clear and striking differences from the healthy and the pseudo-lesioned connectomes. With regard to global hubs, the Real-Lesion Group exhibited a larger number of “new” global hubs. These correspond to nodes with PC levels that qualified them as global hubs in the Real-Lesion Group, although they had not served in that role in the Pseudo-Lesioned or the Healthy Control Group (HC). In terms of location, these new global hubs were concentrated in the same posterior parietal areas as in the HC and the pseudo-lesions (Fig. 8, left) although with greater anterior and ventral extension. With regard to local hubs, we found that the newly recruited hubs were especially concentrated in the contra-lesional right hemisphere (Fig. 8c, right). These findings document that *hub re-definition* is at least one aspect of the long-term re-organization that may follow focal lesions, further underscoring the point that re-organization cannot be understood as simple node subtraction. Future work on this topic should go beyond documenting these differences between real and pseudo-lesioned networks and seek to understand the bases of these long-term changes in nodal characteristics and neurotopography.

### Modelling and understanding dynamic, longitudinal re-organization

Our findings highlight that while there may be acute changes to the functional connectome due to node subtraction, there is also long-term re-organization that occurs in response to the subtraction. This re-organization is presumably directed at restoring brain function in the most optimal manner given: (a) the constraints and limitations created by the node subtraction, (b) the dynamic neural mechanisms that influence network change and (c) experiential and environmental factors that include rehabilitation, environmental stimulation, and so on. The brain’s response to a focal lesion is a dynamic process that unfolds over time and longitudinal network changes should be expected.

Only a very small number of simulation studies have specifically considered the dynamic longitudinal trajectory of neural network development and recovery. For example, Stam et al.^20^ who were primarily interested in the evolution/development of hierarchically structured network organization, posited and modelled two fundamental processes: growth dependent plasticity (GDP) and synchronization dependent plasticity (SDP). GDP was driven by distance-dependent decay, which has the effect of strengthening connections between nearby areas, while SDP is a form of Hebbian learning which strengthens connections between areas with synchronized activity. To emphasize the relevance of these two types of principles, they pointed out that “dynamic processes change connectivity, connectivity constrains dynamics” (p. 2). In addition to examining the role of these principles in network evolution they simulated their role in recovery from focal lesions. Those simulations demonstrated great network resilience and recovery over time and, on that basis, Stam et al.^20^ suggested that network recovery may mimic network development. In another work, Stam et al.^45^ proposed two types of network re-organization such that in the acute phase there is a shift from local to global processing, whereas the opposite occurs in the chronic phase. This would be consistent with reports of low modularity in the acute post-stroke phase followed by increased modularity at later post-stroke time-points^12^ or in response to treatment^11,13^. These observations underscore that future simulation work and network analyses of simulated and real lesions will need to investigate the dynamic long-term changes that are triggered by the focal lesion and the initial node subtraction.

### Task-based vs. rest-state functional connectivity

One major difference between the current study and much of the work in the literature is that rather than using resting-state functional connectivity (RSFC) we examined functional connectivity (FC) obtained from task-based fMRI (after removing task-related activation)—sometimes referred to as “background connectivity” (e.g.^28^). As we mentioned in the Introduction, several studies have looked into the relationship between background connectivity and RSFC, and concluded that they were qualitatively similar^31–34^. For instance, Cole et al.^31^ systematically compared RSFC and task-based FC and concluded that both types of FC were governed by a shared intrinsic organization, with subtle task-related alterations. Moreover, the same “unexpected” relationship between *modularity* and hub damage in pseudo- and real-lesions that we have documented in this paper was also reported in an RSFC study^2^, indicating that our findings are not likely to be specific to the tasked-based FC that we used. Nonetheless, it will be important to validate in the future whether all of the effects we have reported are also observed for RSFC in the same and other cohorts. This would help to elucidate the possibility that brain connectivity associated with different cognitive processes may make different contributions to network re-organization after brain damage.

### Similar global *modularity* between the Healthy, Pseudo-, and Real-Lesion connectomes

We found that, although *modularity* levels differed between Pseudo- and Real-Lesion Groups, in both cases *modularity* levels were comparable to those of healthy controls (Fig. 6a, “Modularity in real and pseudo-lesion”). This raises various questions that we discuss here.

The finding that the Pseudo-Lesion Group did not differ from the Healthy Control Group may seem, at first glance, to be surprising. However, first, it is important to note that the pseudo-lesions used in the current investigation were generated by deleting the actually lesioned nodes identified in the actual stroke lesions and, therefore, affected very different locations compared to the simulated targeted lesions, which did have significant effects on *modularity* (Fig. 5). The pseudo-lesions reflected the typical damage pattern of unilateral middle cerebral artery (MCA) strokes, with most of the lesions centered in the left perisylvian areas including the ventral pre- and post-central gyrus and insula (Supp. Fig. 1). In effect, the pseudo-lesions resemble “random attacks” against which modular organizations have been shown to be highly resilient^19,25,26,46^. For example, Alstott et al.^19^ showed in simulated connectomes that random (rather than targeted) node removal did not affect global network integrity (measured by *global efficiency, average path length*, etc.) until a very large proportion of the nodes had been deleted (see also^25^ for similar results). Relatedly, the left perisylvian region was not the main site for hubs in healthy controls (Fig. 8a), and hence the pseudo-lesions would not have been expected to strongly affect *modularity*.

The Real-Lesion Group (chronic stage, 1–10 years post-stroke onset) also showed comparable levels of *modularity* as the Healthy Control Group (Fig. 6a), although its *modularity* levels were statistically lower than those of the Pseudo-Lesion Group. This result is consistent with the findings in^12^, a longitudinal study that found that *modularity* was initially lower than in healthy controls at the sub-acute post-stroke stage (< 2 weeks), and returned to normal after 3 months. However, although the previous argument regarding random attacks also applies to the Real Lesion Group, we see that, in contrast to the Pseudo-Lesion Group, the distributions of local and global hubs were considerably affected in the Real-Lesion Group (Fig. 8), illustrating that similar levels of global *modularity* can be achieved by different nodal connectivity distributions. The lesson is that findings of similar global values should be interpreted with that understanding. Notwithstanding this point, the finding of comparable global *modularity* values between the Pseudo- and the Real-Lesion Group raises the possibility that the re-organization that we have documented represents the brain’s attempt to achieve a certain degree of global modularity that is optimal for a specific task (e.g.^47,48^). Further understanding of this possibility will require research examining similarities and differences in modular organization following lesions that affect different cognitive domains and lesion locations.

Finally, it is important to emphasize that although global *modularity* may be robust to focal damage, it does not preclude that disruption at the level of local modules can seriously affect the cognitive function(s) the module carries out and can produce long-lasting domain-specific deficits. In a previous study from our lab that examined treatment-induced recovery in a subset of the current stroke group^13^, we found that both the severity of their dysgraphia and their treatment-based improvements in spelling were specifically associated with the functional connectivity properties of the ventral occipital-temporal (VOT) area, corresponding to Cluster #8 in this study (Fig. 3, dark red), an area that forms a key part of the spelling network^49,50^.

### Clinical significance of lesion simulation

Recently, lesion simulation approaches have been used in clinical neuroscience to inform treatment plans and prognoses. One field in which lesion simulation has gained attention is epilepsy. Drug-resistant epilepsy often requires resection of the epileptic focus, yet the safety, precision, and efficacy of the procedure have long been challenging. In recent years, researchers have shown that simulating the effects of resection with sophisticated resection-simulation modelling can retrospectively predict surgery outcomes and may help develop more accurate personalized surgical plans in the future (e.g.^51–53^. Also see^54^ for similar methodology for tumor resection). In stroke recovery, where the goal has been to understand the neural responses to the lesions, only a few studies have directly focused on clinical applications of lesion simulation. Some of these studies have used The Virtual Brain (TVB, www.thevirtualbrain.org) platform that provides modelling methods to generate simulated functional connectivity incorporating dynamic mechanisms^55–57^, offering a way to investigate the underlying neural mechanisms of post-stroke re-organization. For instance, in the context of pre/peri-natal stroke, on the basis of dynamic simulation modelling, Adhikari et al.^51^ argued that early injury might not have sustained consequences and, therefore, that individuals with early stroke might achieve full functional recovery. Using a similar methodology, Falcon et al.^57^ found that in adult chronic stroke, the functional connectome could be best explained by higher values of the “local dynamics” parameter which were related to better treatment-induced motor recovery outcomes. This work is still in early stages, however, and much more empirical research is needed to make progress on the instantiation of the dynamic models and the interpretation of findings. In sum, the dynamic re-organization mechanisms involved in stroke recovery are still largely unexamined, and identifying the mechanisms that govern longitudinal re-organization at different stages (acute, subacute and chronic) can be expected to benefit stroke rehabilitation, including the localization of optimal sites for neuro-stimulation intervention (e.g., tDCS, TMS).

### Limitations

#### Defining hubs, modular organization and characterizing the functional connectome

In this study, we examined damage to two types of “hub” regions: global connector hubs quantified by *participation coefficient* (PC), and local provincial hubs quantified by *within-module degree z-score* (WD)^36^. Although these two graph-theoretic measures are widely used, a variety of other centrality measures have also been used in the literature to define hubs such as *degree*, *betweenness*, etc., which measure slightly different aspects of a node’s connectivity profile (see^58^ for a summary of findings with different hub measures). Here we examined “hubs” defined by PC and WD because these two modular organization, which was the focus of the current investigation. Additionally, these two measures were the ones used in the previous studies that motivated the current study^2,27^. We should also point out that the specific *modularity* measure (i.e., *Newman’s Q*) that we used requires defining a reference modular organization in order to identify the degree to which all the nodes are grouped into different modules. This reference modular organization can be identified in a variety of ways and from a variety of sources. Sources can include previous literature, individual participant connectomes or, as in this study, a control group. Although the use of a healthy control connectome for this purpose allowed us to assess the various lesions in a more consistent way, the approach may have certain limitations. More generally, in future work it will be useful to examine whether or not different hub and modularity definitions will reveal similar patterns or will, instead, reveal other aspects of the neural consequences of focal lesions.

#### Characterizing the lesions

In this paper, we have proposed an approach to understanding the long-term functional network consequences of focal lesions that involves comparisons of real to pseudo-lesioned connectomes. The success of this type of endeavor clearly rests on the degree to which the artificial lesions capture the key characteristics of the real lesions that are relevant to functional network connectivity. Following previous work in this area, we have simulated the focal lesions via node subtraction which creates a “neuroantomical match”. However, it is possible that focal lesions in general (or in our sample in particular) are associated with other brain changes that could independently impact the functional network properties. While work on this topic has been extremely scarce, there are some possibilities that are worth discussing. For example, it has been reported that stroke can cause hypoperfusion (e.g.^59^) and changes in the hemodynamic response function of the BOLD signal (e.g.^60,61^). However, it is worth noting this type of physiological change seems to resolve over time. For instance, Siegel et al.^61^ showed widespread hemodynamic lag of the BOLD signals at the subacute stage followed by a rapid normalization within the first 3 months. Further, Thompson et al.^59^ reported that in chronic stroke, hypoperfusion was only seen in perilesional tissue. Therefore, given that the stroke participants in our study were in the chronic stage (1–10 years post-stroke), we do not expect them to exhibit these types of anomalies. Additionally, it is not at all obvious how high-level functional network characteristics (such as the relationship between *modularity* and cortical hub damage (Figs. 6b and 7) would arise from hypoperfusion. Nevertheless, given the complex interplay between neural, vascular, and metabolic factors, understanding the consequences of vascular abnormalities for functional connectivity remains an important direction of future research.

Another area of interest are the white matter abnormalities indicated by white matter hyperintensity seen on T2-weighted scans outside the lesioned area. In principle, it is possible that these types of abnormalities could (in addition to the simulated node subtraction) contribute to characteristics of the functional connectome. White matter hyperintensity (WMH) is considered an indicator of chronic ischemic damage to periventricular and/or subcortical white matter. While mild WMH is not uncommon in an aging population and its clinical relevance has yet to be decided, pathological levels of WHM has been associated with small vessel disease and general cognitive decline (for a review see^43^). While currently it is not known if or how WMH and functional connectivity are related to each other, it is clearly another important area for future research. Given its possible relevance, we did evaluate the potential role of white matter hyperintensities. As we reported in “Evaluation of white matter hyperintensity (WMH)” and “Evaluation of white matter hyperintensity (WMH)”, for each of the participants with stroke, we identified WMHs based on FLAIR scans. We divided participants into two groups corresponding to those with absent/mild and those with moderate/severe WMHs. While, as expected, the two groups did differ in age (with older participants more likely to be in the moderate/severe group), the groups did not differ in terms of *modularity* and the other network measures we examined.

In sum, as we move forward in using computer simulation to further our understanding of the consequences of brain lesion, it will certainly be very important to have a comprehensive characterization of relevant lesion characteristics so that the effects can be accurately simulated.

#### Characteristics and size of participant groups

First, the sizes of the participant groups in this study were modest (25 participants with stroke with 10 and 13 in the healthy control groups). It will be important in future work to have larger sample sizes to better understand the reliability and generalizability of findings. Second, it is, of course, important that control and experimental groups be matched on relevant variables. In this study, importance was given to matching for age and education. However, we would want to be as confident as possible that the reported differences in functional networks between the Real-Lesion and Pseudo-Lesion Groups are attributable to the effects of the lesions and not to the other factors that might distinguish the groups. As we discussed in the last section, although the two groups might have different vascular characteristics, we did not find this to be a likely source of the differences we found between Real and Pseudo-Lesioned Groups. Another potentially confounding factor is sex. In our sample, 36% of the stroke participants were female, while 80% and 77% were female for the two healthy control groups. However, we did not find differences in *modularity* between men and women (“Modularity in real and pseudo-lesion”) and thus it is unlikely that gender differences could have caused the effects we reported involving *modularity*. Nonetheless, it is desirable to obtain more gender balanced cohorts.

Relatedly, it is important to establish that observed differences between groups cannot be explained by inter-individual differences. To that end, in the analyses comparing the Real and Pseudo-Lesion Groups, we characterized the findings of interest (*modularity* and the relationship between *modularity* and PC and WD damage, “Analysis 2: Comparison of pseudo and real lesions: modularity, PC and WD”) in a manner that preserved inter-individual differences across the healthy controls. We then evaluated the statistical significance of the between-group differences taking into account the inter-individual differences. In other words, the between-group differences for the Real and Pseudo-Lesion Groups (e.g., the positive correlation for the Pseudo-Lesion Group between PC damage and *modularity* and the negative correlation for the Real Lesion Group) were consistently observed across the individuals in the Pseudo-Lesion sample (Fig. 6b,c, also see Supplementary Information 3 for validation with a second HC cohort). This analysis approach increases confidence that reported effects are indeed due to the lesions and not to pre-existing inter-individual differences.

## Conclusions

We examined some of the consequences of focal lesions for functional network organization through a series of analyses comparing the characteristics of functional connectomes subjected to real and artificial lesions. We proposed that the brain’s response to focal lesions involves the effects of both node subtraction and dynamic long-term network re-organization. We used pseudo-lesioned connectomes to instantiate the consequences of node subtraction, and our findings that the real and pseudo-lesions differed in a number of respects strongly support the conclusion that the effects of focal lesions extend beyond node subtraction. These results advance our understanding of the consequences of focal lesions and underscore that future simulation work must include principles of dynamic re-organization in order to provide a more adequate model of brain damage and disease. Such future work will allow us to better understand the underlying neural mechanisms of network re-organization observed in different kinds of brain damage, and whether those changes are necessary for restoring network functionality, or are simply an undesirable consequence of brain lesions.

## Supplementary Information

Supplementary Information.

## Data Availability

The data and analysis scripts that support the findings of this study are available on https://osf.io/thjn6.
