# Publisher Correction: Investigating the network consequences of focal brain lesions through comparisons of real and simulated lesions

**DOI:** 10.1038/s41598-021-88884-3

**Published:** 2021-05-06

**Authors:** Yuan Tao, Brenda Rapp

**Affiliations:** Department of Cognitive Science, Johns Hopkins University, Baltimore, USA

Correction to: *Scientific Reports* https://doi.org/10.1038/s41598-021-81107-9, published online 26 January 2021

The original version of this Article contained an error where an incorrect version of Figure 4 was published.

The original Figure 4 and accompanying legend appears below.

**Figure 4:**
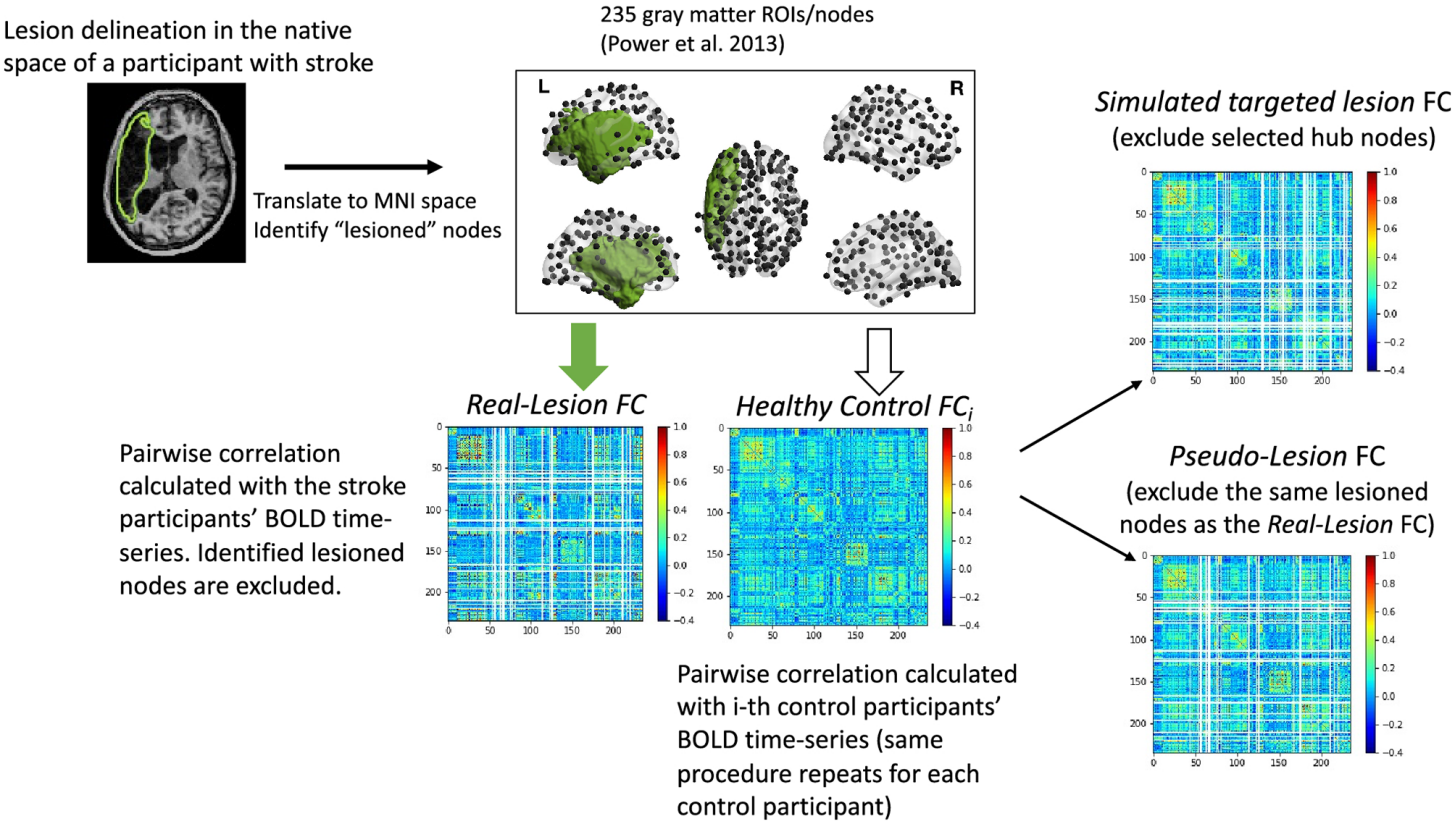
Procedures for generating Real-Lesion, Pseudo-Lesion, and Simulated Targeted-Lesion functional connectomes (FC). For each participant, their functional connectome was calculated based on 235 gray matter ROIs (10 mm-radius spheres), resulting in a 235-by-235 pairwise Pearson correlation matrix (see “Functional connectivity estimation”). **Real-Lesion FC** (bottom-left): for each participant, a lesion mask was first manually drawn on the structural image, then the lesion mask was co-registered to the MNI standard space to identify the “lesioned nodes” (see “Preprocessing” and “Analysis 2: Comparison of pseudo and real lesions: modularity, PC and WD”) and, the correlation values of the lesioned nodes (rows and columns of the matrix, marked as blank in the figure) were excluded from subsequent analyses. **Pseudo-Lesion FC** (bottom center): for each healthy control’s connectome, correlation values corresponding to the “lesioned nodes” were excluded from subsequent analyses. The FC from one healthy control is depicted in which the same rows and columns as in the Real-Lesion FC are left blank. **Simulated Targeted-Lesion FC** (bottom right): for each healthy control’s connectome, correlation values of the identified global/local hub nodes (Fig. 8a) were excluded from subsequent analyses.

The original Article has been corrected.

