# Supplementary material for: Investigating the network consequences of focal brain lesions through comparisons of real and simulated lesions
Source: Sci Rep. 2021 Jan 26;11:2213. doi: 10.1038/s41598-021-81107-9 (PMC7838400; doi:10.1038/s41598-021-81107-9)
Supplement: Supplementary file 1 — Supplementary Information. [file 41598_2021_81107_MOESM1_ESM.pdf]

Investigating the network consequences of focal brain lesions through comparisons of real and simulated lesions

Supplementary Information

Yuan Tao, Brenda Rapp

Department of Cognitive Science, Johns Hopkins University, USA

**Correspondence to yuan.tao@jhu.edu*

Section 1. fMRI task.

The spelling task protocol included a Spelling Probe task and a Case-Verification control task (Rapp & Lipka, 2010[1]) with event-related designs (This experiment was originally designed for a "subtraction-based" GLM analysis and, for this reason, included a control task). Each spelling probe trial consisted of the following sequence of events: 1) task prompt ("Is the letter in the word?") presented both visually and auditorily for 1800ms; (2) a 500ms central fixation cross, (3) an auditory target word plus a variable period of silence depending on the word length, for a total duration of 1500ms during which the fixation cross remained on the screen, (4) A response period consisting of a single visually presented probe letter presented for 1500ms, followed by a 1700ms fixation cross. During this time period, participants responded whether or not the letter was in the spelling of the target word with a button press using with their left index or middle finger. There was a random inter-trial interval (2 to 7.5 seconds) between trials. The Case-Verification trials were identical, except that the task prompt was "Is the letter uppercase?" and participants were instructed to ignore the auditory stimulus and judge the case of the letter with a button press to indicate if the letter was upper or lower case. Each run had 45 trials consisting of 15 Case-Verification and 30 Spelling trials. In order to minimize task-switching costs, 3-6 trials of each condition were presented consecutively in mini-blocks. A total of 4 runs were administered to each participant over two scanning sessions.

Section 2 Lesion overlap.

The lesion masks of the 25 participants with lesion were manually drawn on their high-resolution T1 image and co-registered to the MNI space (preprocessing described in "MRI data analysis: Preprocessing"). The distribution of the lesions followed a typical lesion pattern of middle cerebral artery (MCA) strokes that involved the greatest density of damage in the left insula and superior longitudinal fasciculus.

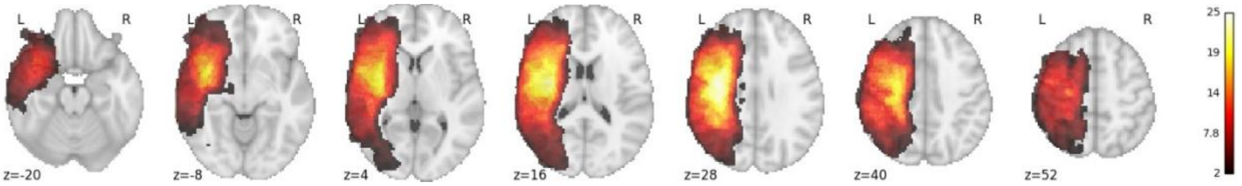

Supplementary Figure 1. Lesion overlap. The color bar indicates the number of participants.

[1] Rapp, B., & Lipka, K. (2011). The literate brain: The relationship between spelling and reading. Journal of cognitive neuroscience, 23(5), 1180-1197.

Section 3. The second healthy control cohort as a validation dataset.

A second healthy control group was used as a validation dataset (HC^v), which consisted of 13 participants (10 females, mean age: 57+/-7.39, years of education: 12-18, three were left-handed). This cohort underwent the same MRI procedure but was administered a modified spelling task, which was the same as the one described in Supplementary Materials Section 1 except that the auditorily presented target words were replaced with black and white line-drawings and participants were instructed to respond on each trial if the visually presented letter was in the spelling of the word depicted in the drawing (or to judge the letter case for Case-Verification trials). Four stroke participants also performed this picture version of the task due to auditory comprehension deficits. All participants were familiarized with the names of the drawings prior to the experiment.

We conducted the same analyses with the second HC group using the same 10-cluster modular organization (Fig. 3 in main text). The effects are the same as observed with the first HC group (see "Results: Validation with a second healthy control cohort" in the main text). Specifically, Targeted-Simulated Lesion produced the expected changes in *modularity* (Supp Fig. 2, left). For Pseudo-Lesion, although *modularity* did not differ from the HC^v Group (Supp Fig. 2, right), it showed the expected relationships with global and local hub damage such that greater global hub (PC) damage led to higher *modularity* and local hub (WD) damage showed the opposite (Supp Fig. 3). At last, the HC^v Group also had similar hub distribution as the first HC group, that is, the global hubs were found in bilateral posterior parietal area and local hubs are found across all modules in both hemispheres (Supp Fig. 4).

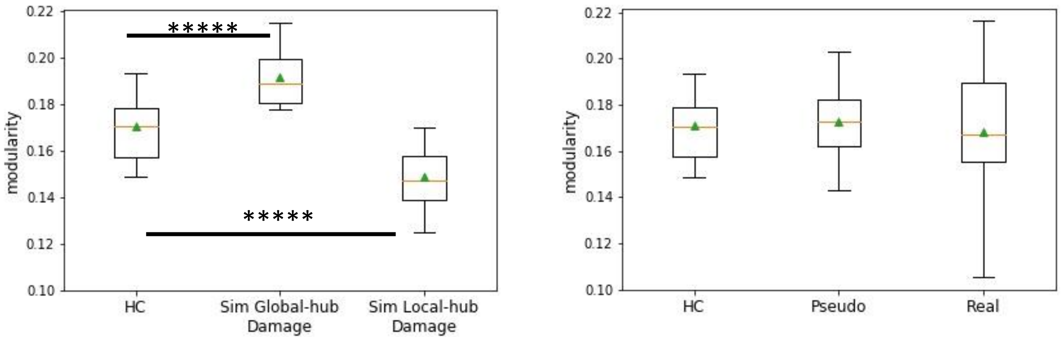

Supplementary Figure 2. Whole-brain *modularity* calculated with the HC^v cohort (N=13) and the corresponding pseudolesions (25-lesion-mask by 13-HC^v = 235). Left: Effect of Targeted Simulated attack, corresponding to Fig. 5 in the main text. Right: Comparison between HC^v, Pseudo-Lesion^v, and the Real-Lesions Group, corresponding to Fig. 6a in the main text (the Real-Lesion Group is the same 25 participants with stroke). The results corresponding to these figures are reported in main text Section 3.4. *****p<10e-06.

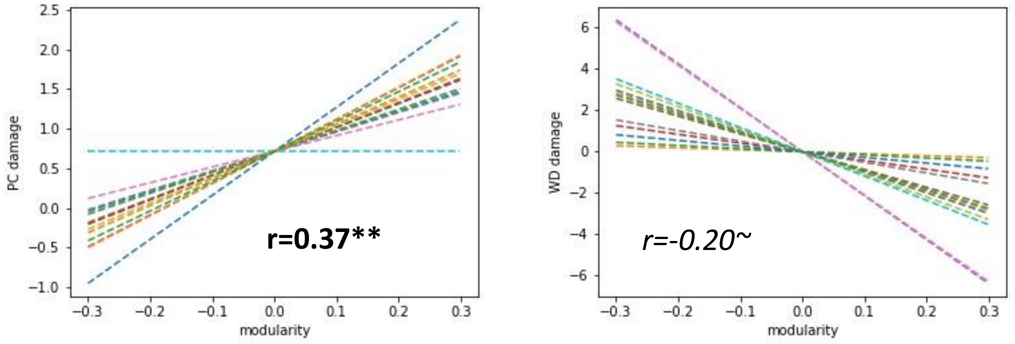

Supplementary Figure 3. Relationship between magnitude of PC (left,) and WD (right) damage and whole-brain *modularity* calculated with the HC^{v} Pseudo-Lesion Group (N=13), corresponding to Fig. 6b in the main text. The HC^{v} Group shows the same effects as the HC Group such that *modularity* is significantly positively correlated with PC damage and negatively correlated with WD damage with marginal significance. The results corresponding to these figures are reported in main text “Results: Validation with a second healthy control cohort”. ~p<0.1, *p<0.05, **p<0.01.

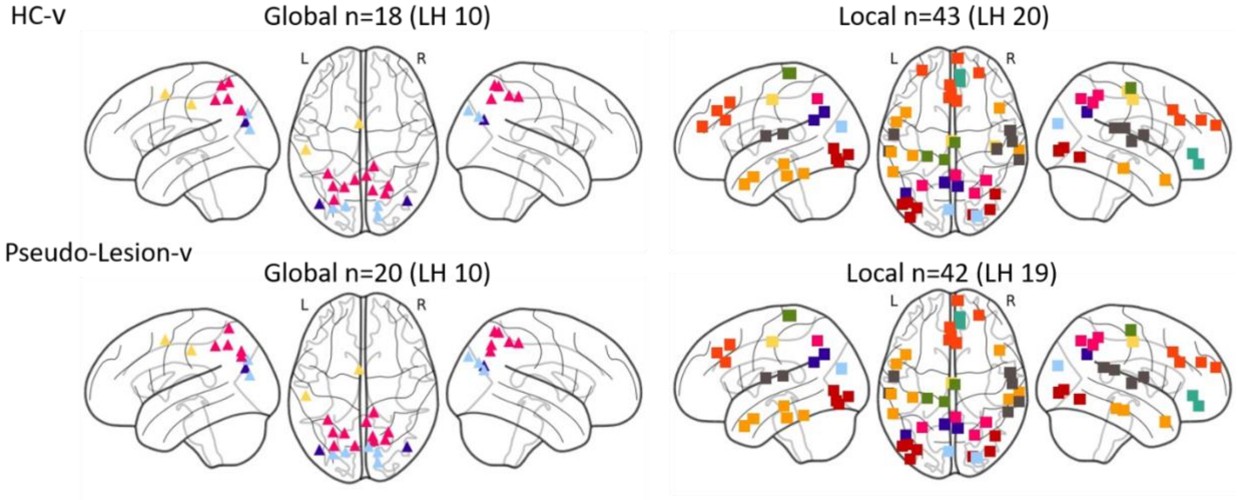

Supplementary figure 4. Global and local hubs identified the HC^{v} Group (N=13) and Pseudo-Lesion Group derived from HC^{v}. These figures correspond to main manuscript Fig. 8a and 8b that are based on the first HC Group. The visualization is created with *Nilearn (https://nilearn.github.io)*.

Section 4. Evaluating different thresholds for identifying the “damaged” nodes.

In the main text, we used a 25% threshold to identify the “damaged” nodes for each lesion mask (“Analysis 2: Comparison of pseudo- and real -lesions: modularity, PC and WD”), that is, for the 235 10mm-radius nodes from the atlas (Powers et al., 2013), nodes that have more than 25% fallen in the lesion mask are considered “damaged” and their correlation values (rows and columns of the correlation matrix) are excluded from subsequence analyses. Here, we evaluated different threshold values to identify the “damaged” nodes. Specifically, we evaluated 10%, 20%, and 30% (higher threshold value, fewer nodes will be considered “damaged”), as some lesion masks do not show any “damaged” nodes with threshold higher than 30%. Analyses described in “Analysis 2: Comparison of pseudo- and real -lesions: modularity, PC and WD” were repeated with those values, and those results corresponding to results described in “Analysis 2: Comparisons between pseudo- and real-lesions modularity, PC and WD” and Fig. 6 in the main text.

The number of “damaged nodes” are overall similar across the threshold values. In the main text, with the value 25%, the mean number of “damaged nodes” across the 25 lesion masks is 16, range 3 to 47. With a lower threshold 10%, the mean number of “damaged nodes” increased to 25, range 9 to 64, and with threshold value 20%, the mean number is 18, range 4 to 52. With a higher threshold 30%, the mean number “damage nodes” decreased 14, range 1 to 46.

Across the threshold values, the results are similar to the main results in the main text. For *modularity* across HC, Pseudo- and Real-Lesion Group, similar to results reported in “Modularity in Real and Pseudo-Lesion” (Fig. 6a) in main text, Real-Lesion tend to show lower *modularity* than Pseudo-Lesion and also lower than HC with a lower threshold 10% (i.e., more nodes are considered damage and excluded). The results are shown in Supp Fig. 5. Likewise, the relationship between *modularity* and global/local hub damage is similar across the thresholds as described in “Different relationships between PC or WD damage and modularity in real compared to pseudo-lesions” in main text and Fig. 6b and 6c, that is, for Pseudo-Lesion, greater global hub (PC) damage leads to higher *modularity* and greater local hub (WD) damage leads to lower *modularity*; by contrast in Real-Lesion, greater global hub damage is associated with lower *modularity* and there is no relationship between local hub damage and *modularity* (Supp Fig. 6).

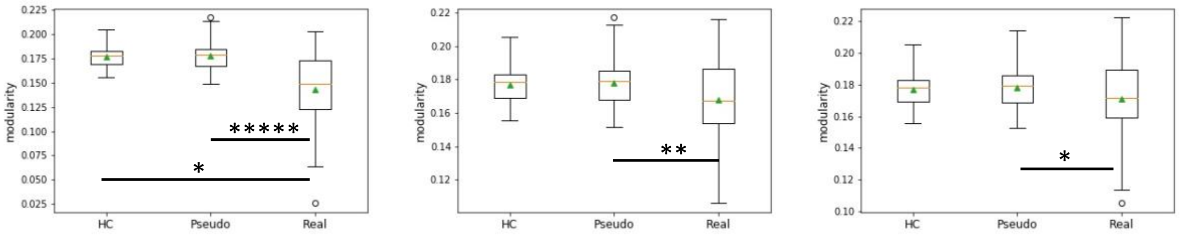

Supplementary Figure 5. Left to right: *Modularity* values with threshold of identifying “damaged nodes” 10%, 20%, 30%. The plots correspond to Fig. 6a in the main text. **p<0.05, **p<0.01, *****p<1e-06*

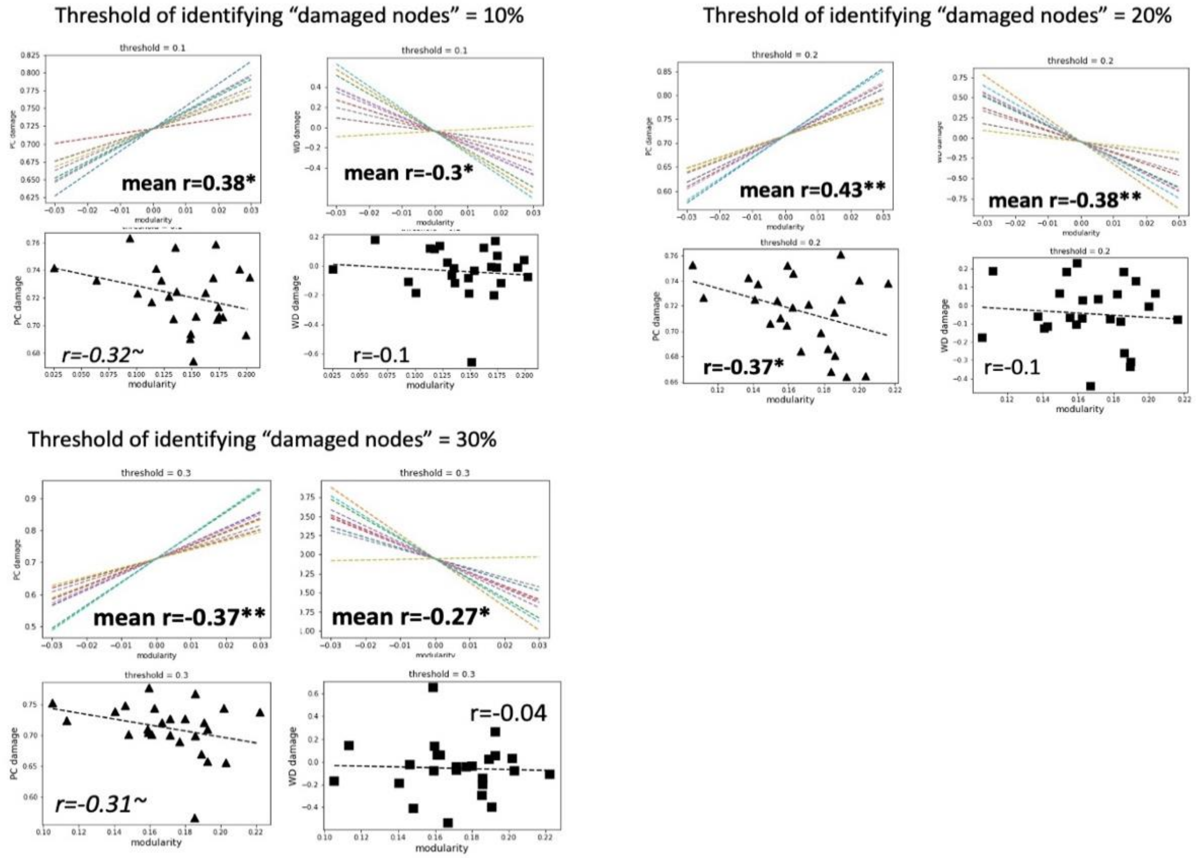

Supplementary Figure 6. Relationship between *modularity* and hub damage across the different threshold values to identify the "damaged" nodes. The figures correspond to Fig. 6b and 6c in the main text: The four panels of each subplot indicate relationship between *modularity* and magnitude of PC (left) and WD damage (right) for the Pseudo-Lesion Group (top) and the Real-Lesion Group (bottom). **p<0.05, **p<0.01, ~p<0.1*.
